# Vacancy defect engineered BiVO_4_ with low-index surfaces for photocatalytic application: a first principles study†

**DOI:** 10.1039/d2ra04890f

**Published:** 2022-11-01

**Authors:** Zhiyuan Zhang, Yingchao Song, Yuqi Xiang, Zhihong Zhu

**Affiliations:** College of Advanced Interdisciplinary Studies & Hunan Provincial Key Laboratory of Novel Nano Optoelectronic Information Materials and Devices, National University of Defense Technology 410073 Changsha Hunan P. R. China zzhwcx@163.com

## Abstract

BiVO_4_ has been widely investigated as a photocatalyst material for water splitting due to its outstanding photocatalytic properties. In order to further improve its photocatalytic efficiency, it is necessary to conduct an in-depth study of improvement strategies, such as defect engineering. By focusing on the (001) and (011) surfaces, we carried out a systematic theoretical research on pristine and defective systems, including Bi, V and O vacancies. Based on density functional theory (DFT), the electronic properties, band alignments and Gibbs free energy of pristine and defective BiVO_4_ have been analyzed. The electronic structures of the (001) and (011) surfaces show different band gaps, and O vacancies make the BiVO_4_ become an n-type semiconductor, while Bi and V vacancies tend to form a p-type semiconductor. Moreover, the band edge positions indicate that holes are indeed easily accumulated on the (011) surface while electrons tend to accumulate on (001). However, the (011) surface with Bi and V vacancies does not have enough oxidation potential to oxidize water. The reaction free energy shows that O and Bi vacancies could lower the overpotential to some extent.

## Abstract 2

We report the OER performance of vacancy defect engineered BiVO_4_ with low-index surfaces. A modulation mechanism can be achieved by the defects.

## Graphical abstract

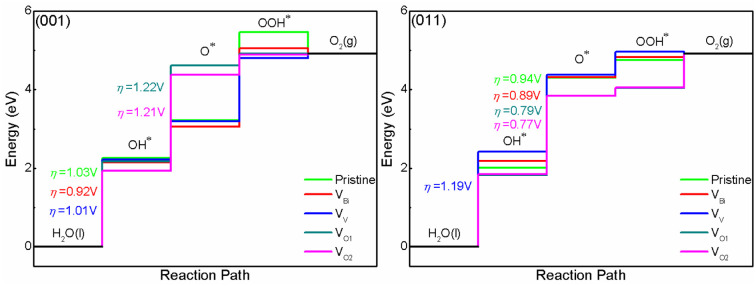

## 1. Introduction

Photocatalytic water splitting into H_2_ or O_2_ has received increasing attention because of its enormous potential to provide a green and renewable way to handle the energy crisis and solve environmental problems.^1–3^ Since the discovery of the first photocatalyst a series of photocatalysts, such as Fe_2_O_3_, TiO_2_, WO_3_, SrTiO_3_ and BiVO_4_, have been investigated for water splitting.^4–8^ Among them, BiVO_4_ has been identified as one of the most promising photocatalysts for water splitting due to its suitable band gap and nontoxic, low-cost and stable nature.^9–12^ The valence band maximum (VBM) of individual BiVO_4_ is well below the redox potential of water, causing an outstanding O_2_ production ability, while its conduction band minimum (CBM) is less positive but very close to the H_2_ evolution potential.^13^ Moreover, BiVO_4_ exhibits a large electron–hole separation yield because of the local polarization caused by the distortion of VO_4_ tetrahedra.^14^ However, the rapid recombination of carriers in this photocatalyst results in a main bottleneck for photocatalytic efficiency, which limits the further application of BiVO_4_ in this field.^15^

In order to further improve the photocatalytic efficiency of BiVO_4_, a series of methods have been researched, including morphology control, elemental doping, composite structure and defect engineering.^16–21^ Recent research shows that defect formation can increase the charge separation efficiency and significantly improve the oxygen evolution reaction (OER) performance of BiVO_4_ for water splitting.^22–27^ The most common type of defect in BiVO_4_ is oxygen vacancies, which can enhance the visible light absorption and promote the charge transfer. Therefore, the photocatalytic efficiency can be improved.^28–30^ However, in some cases the oxygen vacancies might be counterproductive for photocatalytic performance by acting as electron–hole recombination centers.^31^ Furthermore, when the Bi vacancy exists, a higher charge diffusion coefficient can be obtained. And the photocurrent density of this system increases remarkably, even higher than that of previously reported O vacancy engineered BiVO_4_ under the same experimental conditions.^32^ But defects are not always beneficial to improve the photocatalytic performance. V vacancy defect can induce additional states and act as the recombination centers, which cause the decrease of carrier lifetime and photocurrent.^33^

The modulation mechanism of defects has been extensively researched in recent years.^34,35^ In BiVO_4_, the defects affect the electronic structure significantly and thereby the OER performance can be modulated.^36^ However, their exact influence on OER is not fully understood due to studies are still limited and inconclusive.^37,38^ Moreover, for a catalytic process, the reaction occurs on the surface of photocatalyst. Nevertheless most of researches are mainly concentrated on the electronic properties of bulk system with defects.^37–40^ Therefore, it is necessary to conduct in-depth study of the defects under specific surface.

It has been confirmed that the BiVO_4_ has the corner-cut truncated bipyramidal morphology. The (001) surface is the most stable,^27,41–43^ while (011) and (101) comprise the majority of surface area. And the (001), (011), (101) comprise more than 99.3% of the surface area.^44^ Moreover, (011) and (101) can be considered equivalent due to these two surfaces have similar surface energies and morphology.^45^ Therefore, in this work, we focus on the (001) and (011) surfaces. The effect of O vacancy, Bi vacancy and V vacancy engineered BiVO_4_ with representative surface have been investigated based on density functional theory (DFT). We focus on the electronic structure, band edge position and Gibbs free energy of pristine and defective BiVO_4_. The electronic structure of BiVO_4_ has been investigated by calculating the partial density of states (DOS). The band edge positions are the focus of analyzing the photocatalytic mechanism. The photocatalytic activities of pristine and defective BiVO_4_ are studied *via* analysis of the Gibbs free energy. Results show that Bi vacancy might be an effective mean with the lowest overpotential in our calculated systems. And the V vacancy could enhance the overpotential, thus it should be avoided in the experiment.

## 2. Computational details

All the structural optimization and static calculations are based on DFT, as implemented in the Vienna *ab initio* simulation package (VASP).^46,47^ In the calculation, the projector-augmented wave (PAW) method is selected and the generalized gradient approximation (GGA) functional of Perdew, Burke and Ernzerhof (PBE) is used to describe exchange and correlation potentials,^48,49^ here the standard PAW potentials have been chosen. The 5d^10^6s^2^6p^3^ of Bi, 3p^6^3d^4^4s^1^ of V, and 2s^2^2p^4^ of O are treated as the valence electrons. The convergence criterion for energy is 10^−7^ eV for zero-point energy (ZPE) and 10^−5^ for other calculations, 0.01 eV Å^−1^ is selected for the convergence criterion of force. And a cut-off kinetic energy is set to 400 eV for plane wave functions. For BiVO_4_ unit cell and surface system, the Monkhorst–Pack *k*-point grids setting are 7 × 7 × 5 and 4 × 4 × 1 to the first Brillouin zones, respectively. Also *U*_3d_ = 2.7 eV has been used on V atom to correct the self-interaction error.^11^ For the surface system, a vacuum region of 20 Å is added in order to avoid the interactions between layers. And the solvent effect has been considered for the Gibbs free energy calculations as implemented in VASPsol,^50^ the water was selected as solvent here.

## 3. Results and discussion

### 3.1 Bulk geometric optimization

In our calculations, the unit cell of BiVO_4_ has been optimized at first, and here the crystal cell and shape are allowed to be changed. Assuming *c* is the longest axis, the optimized lattice parameters are *a* = 5.17 Å, *b* = 5.16 Å, *c* = 11.76 Å, *α* = 89.999°, *β* = 90.003°, *γ* = 90.145°. The photocatalyst BiVO_4_ exits in monoclinic scheelite (ms-) and tetragonal scheelite (ts-) phases. In the process of optimization based on DFT, the ms-BiVO_4_ (*a* = 5.194 Å, *b* = 5.09 Å, *c* = 11.667 Å, *α* = *β* = 90°, *γ* = 90.4°) would spontaneously transform to ts-BiVO_4_ (*a* = *b* = 5.147 Å, *c* = 11.722 Å, *α* = *β* = *γ* = 90°) when crystal cell and shape are allowed to be changed,^41,51^ therefore, the BiVO_4_ might tend to ts-BiVO_4_ in our calculation, and the lattice constants slightly expand by less than 1% compared with ts-BiVO_4_. Due to ms-BiVO_4_ and ts-BiVO_4_ have a similar structure, both ms-BiVO_4_ and ts-BiVO_4_ can be used for calculating photocatalytic performance, which would not change the important results and conclusions.^52^

### 3.2 Surface geometric structure

**Fig. 1:**
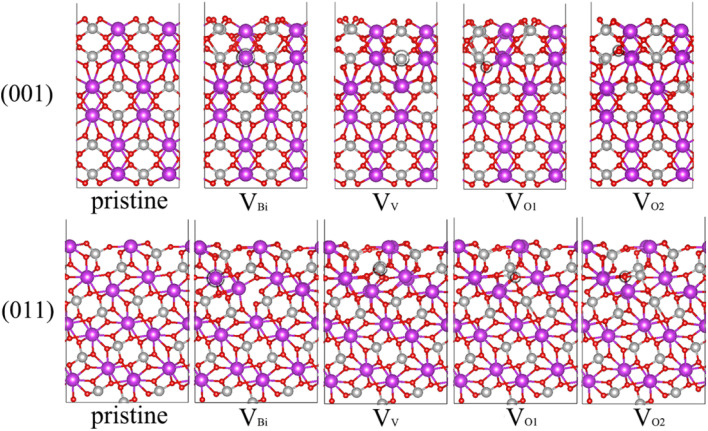
Side view of optimized geometric structures of BiVO_4_ surface. Bi (purple), V (silver) and O (red) atoms are shown in colored spheres. The vacancy site is marked by black circle.

Due to the (001), (011) and (101) surfaces cover most area of BiVO_4_ and (011) and (101) can be considered equivalent,^44,45^ thus we focus on (001) and (011) surfaces. In some previous studies, the *b* is set as the longest axis. In this case, the corresponding surfaces should define (010), (110) and (011). Moreover, there are some studies rotating the BiVO_4_ 90° around the longest axis *c* compared our bulk BiVO_4_, thus (011) should define (101) in these existent studies. For one BiVO_4_ unit cell, it contains one kind of Bi, one kind of V and two kinds of O. Therefore, four possible kinds of vacancy defects of BiVO_4_ are examined, namely, Bi vacancy engineered BiVO_4_ (V_Bi_), V vacancy engineered BiVO_4_ (V_V_), the first kind of O vacancy engineered BiVO_4_ (V_O1_) and the second kind of O vacancy engineered BiVO_4_ (V_O2_), also the pristine BiVO_4_ with (001) and (011) surface are considered here. The unit cell of bulk BiVO_4_ has been cleaved to obtain the (001) and (011) surfaces, then the two slabs has been adjusted to make the thickness of them larger than 15 Å. And the BiVO_4_ (001) and (011) models are constructed by 2 × 2 and 2 × 1 corresponding slabs, respectively. The side and top views of optimized geometric structures are shown in Fig. 1 and S1,† respectively. The vacancy concentration of (001) is 4.2% for V_Bi_ and V_V_, 1.0% for V_O1_ and V_O2_, while that of (011) is 3.6% for V_Bi_, 3.3%for V_V_, 0.9% for V_O1_ and V_O2_. After carefully checking the structures of defective BiVO_4_, it can be seen that the obvious distortion has been formed near the defect, which might modulate the electronic properties and affect the photocatalytic performance of BiVO_4_ significantly.

### 3.3 Electronic structures

**Fig. 2:**
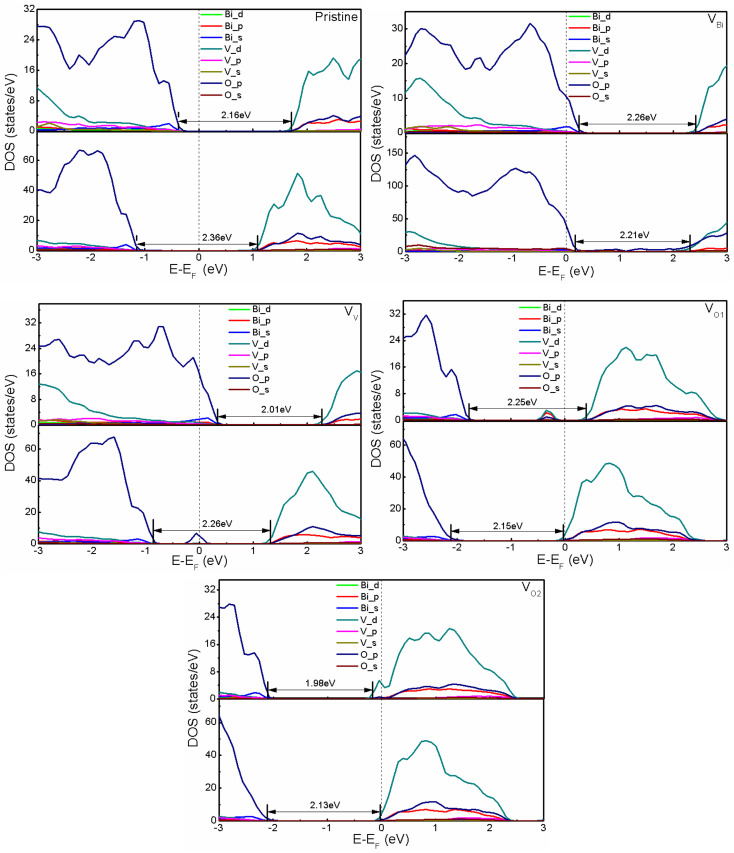
The calculated DOS of pristine and defective BiVO_4_. The upper part and bottom part represent (001) and (011), respectively. The Fermi level is set to zero.

After obtaining the most stable structures, the electronic structure of defective BiVO_4_ (001) and (011) have been investigated by calculating the partial DOS. And the calculated results are shown in Fig. 2. Clearly, the band gaps of pristine BiVO_4_ (001) and (011) are 2.16 eV and 2.36 eV, respectively. This result is consistent with the previous GGA + *U* calculation (2.123/2.083 eV for (001) surface and 2.244/2.250 eV for (011) surface) and LDA + *U* calculation (2.18 eV for (001) surface and 2.38 eV for (011) surface).^45,52^ And the Fermi level of pristine BiVO_4_ (001) is close to the VBM and far from the CBM, while that of pristine BiVO_4_ (011) is almost in the middle of band gap. Here the VBM is mainly populated by O 2p and the CBM is composed of V 3d in both pristine BiVO_4_ (001) and (011) systems. For the band edges of defective BiVO_4_ (001) and (011), also it can be observed VBM and CBM are contributed by O 2p and V 3d, respectively. However, there are still some differences existing in different structures, especially the Fermi level and band gap. It can be seen that V_Bi_ and V_V_ tend to make the pristine BiVO_4_ become a p-type semiconductor, while V_O1_ and V_O2_ tend to form a n-type semiconductor. Generically speaking, O vacancy is easy to form in the process of synthesis, thus the BiVO_4_ is often treated as a n-type semiconductor rather than p-type semiconductor in the experiments. This phenomenon indicates the V_Bi_ and V_V_ could introduce holes while V_O1_ and V_O2_ would introduce electrons, respectively. Moreover, the band gap, the Fermi level and band edge could be controlled by defects, indicating the oxidation and reduction capacity probably can be modulated according to introducing defects. Notably, a peak appears near the middle of band gap for V_V_ with (011) surface and V_O1_ with (001) surface. The peak of V_V_ with (011) is very close to the Fermi level, which might be caused by defect states, and it probably acts as the recombination center, which is not good for photocatalysis to some extent.

### 3.4 Band alignments

**Fig. 3:**
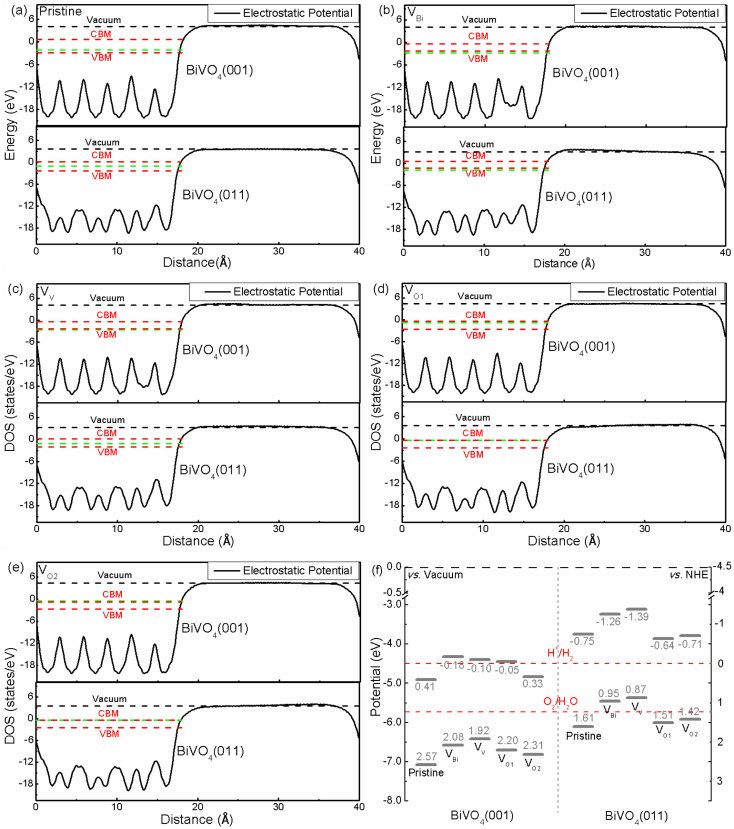
(a)–(e) The relative positions of electrostatic potential of pristine and defective BiVO_4_, (f) band edge potentials for BiVO_4_ (001) and (011). The green dash line represents the Fermi level.

The band edge position is an important role for photocatalytic application. Hence the band edge position of BiVO_4_ has been analyzed based on macroscopic averaging method,^53^ where the electrostatic potential has been chosen as a reference in order to obtain the band edge. The relative positions between the Fermi level and CBM/VBM are obtained according to their individual supercell. The results are shown in Fig. 3(a)–(e). Clearly, the electrostatic potentials of different facets are quite different. And the difference valued between CBM/VBM and vacuum level is 4.91/7.07 eV for pristine BiVO_4_ (001), and 3.75/6.11 eV for pristine BiVO_4_ (011), respectively, meaning the CBM and VBM of pristine BiVO_4_ (011) is higher than that of pristine BiVO_4_ (001). Therefore, the photogenerated electrons on the (011) would migrate to the (001) and the photogenerated holes could transfer from (001) to (011). This is consistent with the experimental results, where the researchers obtained direct evidence that holes are indeed easily accumulated on the (011) surface and electrons tend to accumulate on (001).^54^ Although the average potentials of two facets are dramatically changed, similar behaviors could be found in V_Bi_, V_V_, V_O1_ and V_O2_, where the CBM and VBM of (011) are still higher that of (001). And the charge separation could be promoted near the crystal boundary of these two facets. Under the light irradiation, the photogenerated electrons would migrate to BiVO_4_ (001) from BiVO_4_ (011), and the photogenerated holes would transfer from BiVO_4_ (001) to BiVO_4_ (011) at the same time. Therefore, the BiVO_4_ (001) and BiVO_4_ (011) could be the reduction site and oxidation site, respectively. This result is consistent with the previous experimental results. Moreover, it can be inferred that the V_Bi_, V_V_, V_O1_ and V_O2_ cannot change this situation due to CBM and VBM of BiVO_4_ (011) are still higher than BiVO_4_ (001) after introducing these defects.

Considering the relationship between potentials of normal hydrogen electrode (NHE) and vacuum level (*E*_vacuum_ = −*E*_NHE_ − 4.5 eV), the band edge positions related to H^+^/H_2_ level and O_2_/H_2_O level are plotted, as shown in Fig. 3(f). We can see that the band edge positions are totally different. For (011) surface, the VBM of V_Bi_ and V_V_ are located above the corresponding O_2_/H_2_O potential, indicating the biased voltage is necessary for V_Bi_ and V_V_ to produce O_2_. As for (001) surface, the VBM of pristine BiVO_4_, V_Bi_, V_V_, V_O1_ and V_O2_ are below the O_2_/H_2_O level, meaning these systems have ability to produce O_2_ without biased voltage. Totally speaking, for (011) surface, V_Bi_ and V_V_ might not be a good way for photocatalytic application due to that the biased voltage must be added.

### 3.5 Overpotential

In order to further investigate the photocatalytic activity, the OER performance of pristine BiVO_4_, V_Bi_, V_V_, V_O1_ and V_O2_ with (001) and (011) surfaces have been analyzed according to overpotential. Here the computational hydrogen electrode (CHE) model has been adopted.^55^ Generally speaking, there are four steps in the OER process, and each step contains one electron transfer. For the first step, the H_2_O could be dissociated at the Bi site under the influence of photogenerated hole, and then a proton would be released and the OH radical would be formed. The second step is the reaction that OH radical releases another a proton and forms the O with the interaction of photogenerated hole. Then, the generated O would be combined with adjacent H_2_O, forming the OOH radical and releasing a proton. At last, the OOH radical would further release a proton and form the O_2_, then the O_2_ leaves the surface. The optimal OER reaction path could be described as:

1 H_2_O + * ⇌ OH* + H^+^ + e^−^

2 OH* ⇌ O* + H^+^ + e^−^

3 O* + H_2_O ⇌ OOH* + H^+^ + e^−^

4 OOH* ⇌ O_2_ + H^+^ + e^−^

where * stands for the reaction site of photocatalyst, and H*, OH*, O* and OOH* refer to adsorbed intermediates in the OER process. The decisive role for overpotential (*η*) is determined by the largest Gibbs free energy change (Δ*G*) among four reaction steps:

5 *η* = −max[|Δ*G*_OH*_|, |Δ*G*_O*_ − Δ*G*_OH*_|, |Δ*G*_OOH*_ − Δ*G*_O*_|, |4.92 − Δ*G*_OOH*_|]/e − 1.23

Without the biased voltage, the Δ*G* can be obtained by calculating the difference of Gibbs free energy between product and reactant:

6 Δ*G* = Δ*E* + Δ*E*_ZPE_ − *T*Δ*S*

in which the Δ*E* refers to adsorption energy, Δ*E*_ZPE_ and Δ*S* stand for ZPE and entropy at the specific temperature *T*, respectively. The relationship of Gibbs free energy in the CHE model meet the conditions:

7 *G*(H^+^) + *G*(e^−^) = 1/2*G*(H_2_)

8 *G*(H^+^) + *G*(OH^−^) = *G*(H_2_O)

9 2*G*(H_2_) + *G*(O_2_) − 2*G*(H_2_O) = 4.92 eV

In our calculation, the free energy of O_2_ is obtained by eqn (9) rather than DFT because of the large error for calculating O_2_ in VASP program. The calculated free energy of H_2_, O_2_ and H_2_O are listed in Table S1.†

**Fig. 4:**
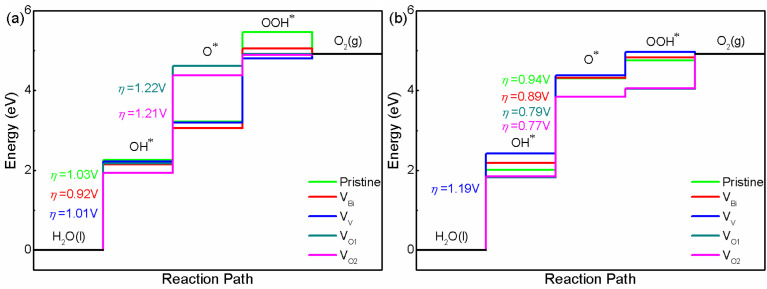
The calculated OER free energy of (a) BiVO_4_ (001) and (b) BiVO_4_ (011).

After obtaining the free energy of H_2_, O_2_ and H_2_O, the adsorbed intermediates have been investigated. The structures of adsorbed intermediates (OH*, O* and OOH*) of pristine BiVO_4_, V_Bi_, V_V_, V_O1_ and V_O2_ with (001) and (011) surface are plotted in Fig. S2 and S3.† And the calculated total energy, ZPE and entropy for all the structures are shown in Table S2.† For these structures, BiVO_4_ substrate has been fixed and the adsorbed atom has been optimized at first, then the adsorbed atom and the top layer of substrate are relaxed together. The calculated results about overpotential of pristine and defective BiVO_4_ are shown in Fig. 4(a) and (b). For all the systems, the limiting step is the first step or the second step. Clearly, V_Bi_, V_V_, V_O1_ and V_O2_ could impact the free energy greatly. Here we focus on pristine BiVO_4_ at first. It can be found that (001) has a lower overpotential compared with (011). Hence, (011) is more active than (001) for OER. For this model, the overpotential is close to previous calculated studies, such as PBE0 with implicit solvent model calculation (1.2 V for (001) and 0.9 V for (011) surface) and GGA calculation (1.42 V for (001) and 1.14 V for (011) surface).^44,56^ Then we turn to defective BiVO_4_. For (001), it can be seen that the V_Bi_, V_V_, V_O1_ and V_O2_ could lower the free energy for forming OH* and OOH*. These two steps consist of one H removal, which is more efficient in all of the reaction steps happening on a surface. This phenomenon might be because the defects introduce additional holes or electrons near the Fermi level (the V_Bi_ and V_V_ introduce holes while V_O1_ and V_O2_ introduce electrons). These carriers could promote the charge transfer and lower the free energy. In the process of forming O*, V_Bi_, V_V_ show a lower free energy compared with pristine BiVO_4_, while V_O1_ and V_O2_ show a higher free energy. This phenomenon might due to the pure O tend to be combined with holes rather than electrons for this surface, in these system, the V_Bi_, V_V_ introduce additional holes while V_O1_ and V_O2_ introduce electrons. Totally speaking, the V_O1_ and V_O2_ reduce the photocatalytic activity due to a large Δ*G*, while V_V_ only lower the overpotential slightly and the difference could be negligible, thus V_V_ probably do not affect the photocatalytic performance for this surface. And V_Bi_ could improve the photocatalytic performance to some extent. Then we turn to OER on the (011) surface, and here an analogous set of calculations was performed. The results show that the pristine (011) surface is the more efficient than (001), and the second step becomes the potential limiting step. With respect to the pristine surface, the free energy of defective BiVO_4_ with (011) surface show a different variation trend compared with (001). It can be seen that the V_O1_ and V_O2_ lower the free energy while V_Bi_ and V_V_ enhance the free energy for forming OH*, O* and OOH*. This phenomenon is completely different compared with (001), indicating the OH, O and OOH radicals might tend to be combined with electrons rather than holes for this surface. For (011) surface, we can see that the OER efficiency is reduced when the V_V_ is introduced, while it can be improved when the V_Bi_, V_O1_ and V_O2_ is introduced. The overpotential of V_O1_ and V_O2_ shows a similar behavior with some previous studies,^57,58^ where the O vacancies enhance the overpotential for (001). However, there are also some previous studies showing a different situation, where the O vacancies lower the overpotential for (001) and enhance the overpotential for (011).^44^ This phenomenon probably due to the different location of O vacancies, thus it can be inferred that O vacancies is an effective but uncontrollable method for OER. In our calculated systems, the V_V_ is not beneficial to enhance OER performance while V_Bi_ would contribute to improving OER performance. The calculated results is consistent with the previous experimental results.^32,33^ Therefore, the V_V_ should be avoided in the experiment. And the V_Bi_ should be adopted to improve the OER performance.

### 3.6 Origins of free energy change

**Fig. 5:**
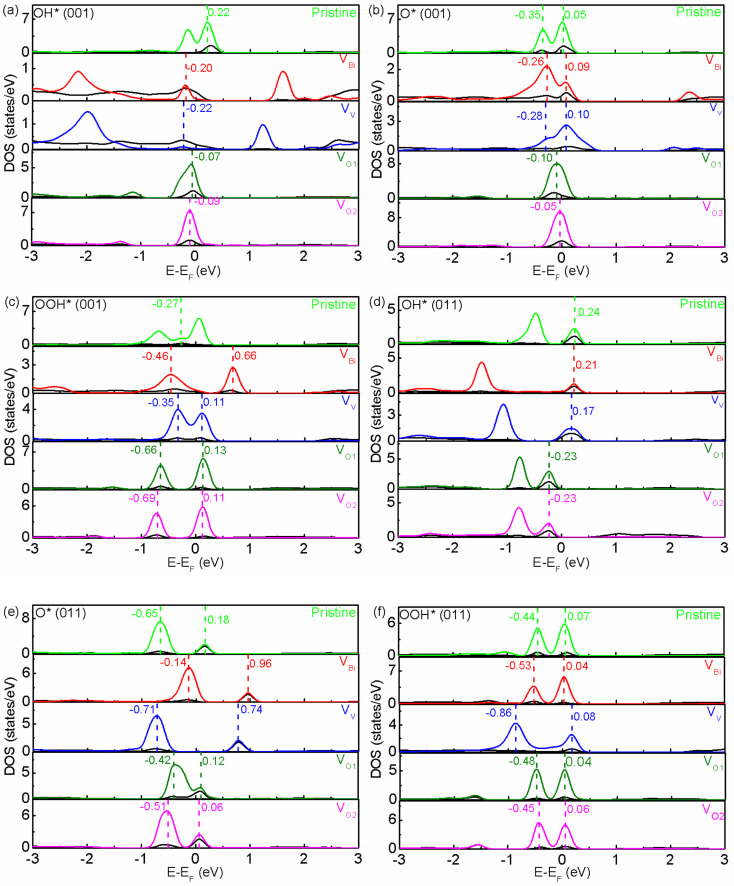
(a)–(f) The partial DOS of pristine and defective BiVO_4_. The black and multicoloured line represent the partial DOS of active site and adsorbate, respectively.

In order to reveal the origins of free energy change, the partial DOS of active sites and adsorbate have been examined. In a chemical reactions, the frontier molecular orbitals in the proximity of Fermi level play an important role.^59^ As show in Fig. 5(a)–(f), it can be seen there are two or one hybridized states between active site and adsorbate for O* and OOH*. Totally speaking, two hybridized states near the Fermi level would have a lower free energy. For (001), the pristine BiVO_4_, V_Bi_ and V_V_ have a much lower free energy compared with V_O1_ and V_O2_ for O*, and pristine BiVO_4_ has a much higher free energy compared with V_Bi_, V_V_, V_O1_ and V_O2_ for OOH*. Moreover, the free energy would increase as the distance between two hybridized states become greater. This phenomenon might due to a larger distance between two hybridized states increases the difficulty in transferring charge and weaken the adsorption. And one hybridized state makes the charge transfer more difficultly and further weaken the adsorption. Similar behavior also can be found in (011) although the exact locations of hybridized states are different. For OH*, there is one hybridized state between active site and adsorbate. And it can be seen the free energy is lower when the hybridized state is below the Fermi level. Moreover, it can be found the free energy is related to the distance between the Fermi level and the hybridized state. When the hybridized state is below the Fermi level, the free energy decrease with a smaller distance. However, when the hybridized state is above the Fermi level, the free energy is unexpectedly enhanced with a smaller distance. This result is difficult to explain and ought to be investigated in more detail in the future. We guess this phenomenon is due to positive charges have already accumulated on the active site, which would promote holes transfer and inhibit electrons transfer between active site and adsorbate. Therefore, the free energy could be modulated. The Bader charge analysis for active site has been shown in Fig. S4,†^60^ it is clearly that positive charges have accumulated on the active site. After inducing defects, the charge distribution on this site has a slight, not obvious change. According to the results of hybridized states and Bader charge, it can be inferred defects mainly affect the hybridized states between active site and adsorbate, thus the free energy for each step can be adjusted and the overpotential can be changed.

## 4. Conclusions

In summary, we have carried out a comprehensive periodic density functional theory (DFT) simulations for the defective BiVO_4_ with (001) and (011) surfaces to improve its photocatalytic performance. It is found that defects have a great effect on BiVO_4_. V_V_ with (011) surface and V_O1_ with (001) surface create defect states near the middle of band gap, which might not be good for photocatalysis to some extent. The band edge position indicates that V_Bi_ and V_V_ with (011) cannot produce O_2_ without biased voltage. According to modeling all of the reaction intermediates for different water oxidation mechanisms, we have shown that the most favorable photocatalytic process on BiVO_4_ is the (011) surface. The defects could change the overpotential greatly, and V_Bi_, V_O1_ and V_O2_ exhibit the best photocatalytic activity due to its lower overpotential in our calculated systems. Moreover, the defects have a great effect on hybridized state between active site and adsorbate. By controlling the exposed surface facet and the vacancy content, OER performance can be improved, which is important for the design of novel photocatalyst.

## Conflicts of interest

There are no conflicts of interest to declare.

## Supplementary Material
